# A mixed-method study to inform the design of a video observed therapy app to monitor individuals with TB in the Dominican Republic

**DOI:** 10.3389/fpubh.2025.1529687

**Published:** 2025-06-06

**Authors:** Julio Arturo Canario-Guzmán, Ricardo Elias-Melgen, Eddys Rafael Mendoza, Luis Felipe Arias, Roberto Espinal, Jeannette Báez, Sarah Iribarren

**Affiliations:** ^1^Centro Nacional de Investigaciones en Salud Materno Infantil Dr. Hugo Mendoza (CENISMI), Santo Domingo, Dominican Republic; ^2^University of Washington, Seattle, WA, United States

**Keywords:** video observed therapy, digital adherence technologies, Tuberculosis, patient monitoring, Dominican Republic

## Abstract

**Introduction:**

Video Observed-Therapy, a digital adherence technologies (DAT), could improve patients’ adherence to tuberculosis (TB) treatment. However, countries with high TB burden countries, such as the Dominican Republic, struggle to adopt DAT as a control and prevention tool to improve clinical outcomes. Objectives: To describe patients’ knowledge, attitudes and behaviors towards digital technologies for TB control and prevention in the Dominican Republic.

**Methods:**

A mixed-method study was implemented by organizing two Focus Group Discussions (FGDs) and a survey with 137 TB patients. Qualitative data were coded using the Integrated Behavior Model (IBM) and descriptive statistics were used to analyze knowledge, attitudes, practices, and preferences. Data integration was achieved by comparing convergent and divergent results.

**Results:**

Participants expressed a positive attitude towards using DAT to facilitate communication with health personnel to support treatment adherence. Participants noted the benefit of having an application that facilitates this process while safeguarding data privacy.

**Conclusion:**

This study demonstrates positive attitudes among individuals with TB toward adopting digital tools in their follow-up. Current practices suggest that intent to use DAT is prevalent, emphasizing the need to fully transition from intention to adoption of DAT to improve TB treatment outcomes.

## 1 Introduction

The World Health Organization (WHO) and global partners recognize the critical role of digital health in advancing the global response to tuberculosis (TB) and have designed a comprehensive digital health agenda (1–4). Despite these efforts, the adoption and integration of digital adherence technologies (DAT) in the health sector, particularly in low- and middle-income countries remains challenging (5). The Dominican Republic (DR), with a high TB incidence rate, reported approximately 45 [33–57] new TB cases per 100,000 inhabitants in 2021 (6).

To reduce the burden of TB in the DR, the National TB Program (NTBP) of the Ministry of Public Health (MoPH) implements the Directly Observed Treatment Short-Course (DOTS) strategy. However, in 2020, 16% of the TB patients who started DOTS did not complete their treatment (7). Several factors contribute to incomplete treatment including difficult access to healthcare, patient knowledge, poverty, drug use, comorbid illnesses, treatment-related adverse events, poor patient-provider communication, and self-treatment (8–10).

To achieve WHO target of 90% treatment success rate (11), various initiatives have been made. These efforts include shortening treatment duration (12), improving medication adherence, integrating digital technologies and health behavior models to increase effectiveness in changing behavior and improving medication. Digital technologies (short message services -SMSs- via mobile phones) and, more specifically, digital adherence technologies (DATs) tools designed to support individuals with TB with taking their medication (medication sleeves; smart pill boxes; video-observed treatment) (13–15).

From the available alternatives to increase treatment adherence, we proposed the use of Video Observed Therapy (VOT). In the Dominican Republic, the use of messaging and video application is widespread and widely accepted. Also, VOT could address barriers with DOTS, such as travel time and related costs (16–21). TB is considered a disease of the poverty, so patients can benefit from reducing the number of visits to the clinic. VOT as intervention has evidence of improvement medication adherence (22).

At the inception of this study in 2018, VOT had not been implemented in the DR. The onset of the COVID-19 pandemic disrupted TB control efforts but accelerated global adoption of digital tools in TB programs (5, 23). In response, the DR developed a new protocol to facilitate the use of DAT for remote TB patient follow-up. Extending drug supply periods and implementing remote monitoring and support proactively can mitigate the risk of patient loss to follow-up (24–27). The overall objective of this study was to explore and describe TB patient attitudes and behavior towards digital technologies for TB control and prevention.

## 2 Methods

### 2.1 Study design

A convergent mixed-method design was used to pursue the aim of this study. Qualitative and quantitative components were combined to explore and describe TB patients’ attitudes and behavior, regarding using digital technologies in their TB care and management. Focus group discussions (FGDs) were held with nine individuals with TB, and 137 were surveyed between September and January 2021 in Santo Domingo, Dominican Republic. Ethical approval was obtained from the Research Ethics Committee at the Centro Nacional de Investigaciones en Salud Materno Infantil Dr. Hugo Mendoza (CENISMI) (#31082020). Written informed consent was obtained from all study participants. Confidentiality and data protection measures were undertaken by using encrypted laptops and files with restricted access to the study team and PI.

### 2.2 Qualitative component: participant focus group discussions

A FGD guide with open-ended questions was developed based on the Integrated Behavioral Model (IBM) (28) constructs, which combines constructs from the Theory of Reasoned Action (29) and Planned Behavior (30). Questions include, for example, have you ever used a telephone or a computer with the Internet to search for health information or monitor your health status? What do you like/dislike about the idea of reviewing a video recording of the patient taking medication at home and sending it to the doctor? We aimed to explore participants’ knowledge, attitudes, practices, and preferences for using digital technologies during their treatment. The questionnaire was discussed between the research team, and comments on content validation and language and cultural appropriateness of the questions. The questionnaire passed by different rounds of revision until a final version was agreed upon by the researchers. The questionnaire is in the Annex section.

The FGDs were conducted in TB Units in two health centers in the province of Santo Domingo (Boca Chica and Villa Mella). Inclusion criteria were being bacteriologically confirmed with TB, 18 years of age and older, who had initiated or recently completed treatment (within the last 3 months). Convenience sampling was used. Healthcare workers from the TB units recruited participants consecutively in person as they came in for follow-up appointments. If interested in participating, they were connected with a research team member who facilitated the informed consent process and scheduling the FGDs. Each FGDs session lasted approximately 1 h and was audio-recorded.

### 2.3 Quantitative component: survey

For the quantitative component, identical inclusion/exclusion criteria were applied. Participants were recruited from eight TB units across the province of Santo Domingo and the National District with the highest cases of TB in the country. Health care workers at TB units recruited participants. The survey was administered in person. The survey consisted of 58 questions in 6 sections: (a) sociodemographic, clinical, and adherence characteristics, (b) knowledge, (c) attitude, (d) practices, (e) access to health care, and (f) economic consequences. The questionnaire included questions on preferences for TB digital treatment monitoring. Data from medical records was collected to describe the clinical characteristics of the participants.

### 2.4 Analysis

FGD sessions were transcribed verbatim and independently coded by two research team members using practical thematic analysis that uses both deductive and inductive analysis. Categories and themes were developed *a priori* based on the IBM constructs to create a structured categorization matrix. Inductive codes were also developed by the two members, who meet as a team regularly to review codes and ensure consistency in reaching a consensus collaboratively. The data was analyzed using thematic analysis. The steps Saunders et al. suggested to conduct a practical thematic analysis were followed (31).

Statistical analysis was performed in the quantitative study phase using SPSS 20.0 Software. Descriptive statistics are presented in percentages, median, max and min values. The data from continuous variables (age, schooling, etc.) are presented as the median value plus the minimum and maximum values. Demographic data are presented as frequency and percentage. Quantitative and qualitative data were analyzed separately and compared to explore convergent and divergent results. The Good Reporting of a Mixed Methods Study (GRAMMS) guidelines were used to guide the reporting of this study (31).

## 3 Results

### 3.1 Quantitative findings

#### 3.1.1 Survey participant characteristics

137 TB individuals with TB participated in the study, with 40 (*n* = 29.2) in the National District and 97 (*n* = 70.8) in Santo Domingo. Among the participants, 75 (54%) were male. Most survey participants identified as Dominican (*n* = 133, 97.1%), three Haitians (*n* = 3, 2.2%), and one Venezuelan (*n* = 1, 0.7%). Most reported being single (*n* = 78, 56.9%) and with a partner (*n* = 47, 34.3%). Most participants reported literacy skills, with 97.1% (*n* = 133) being able to read and write. Regarding education, 33% (*n* = 45) had a primary school level, nearly 50% (*n* = 69) completed secondary school, and 12% (*n* = 16) attended university (Table 1).

**Table 1:** Sociodemographic and clinical characteristics of the TB patients who were included in the study, Santo Domingo, 2019 (*n* = 137).

Variables	*n* = 137	%
Sex		
Male Female	75	54.7
Province		
Distrito Nacional	40	29.2
Santo Domingo	97	70.8
Age—mean age (+ − SD) Age (in years)	xx	
18–24	31	22.63
25–34	35	25.55
35–44	24	17.52
45–54	27	19.71
55–64	12	8.76
>65	8	5.84
Nationality		
Dominican	133	97.1
Haitian	3	2.2
Venezuelan	1	
Schooling level		
Primary school	50	35.5
Secondary school	66	48.2
University	18	13.1
None	3	2.2
Literacy (know how to read and write)	133	97.1
Place where the patients get the medication		
At the healthcare center	127	92.7
At home	10	7.3
Clinical characteristics		
Newly admitted patients	122	81.8
Patients recovered after dropout	10	7.3
Relapse	10	7.3
Previously treated patients	5	3.6
Mean time in the treatment (in month), M (SD)	4.7 (3.24)	
Irregular or nonadherent	22	16.1
Comorbidities		
Had at least one comorbidity	47	35.0
Diabetes Mellitus	16	11.6
HIV	15	10.9
Other	17	12.5
Substance use		
Alcohol use	12	8.8
Tobacco use	8	5.8
Drugs use	2	1.5
Have a health insurance	117	85.4
Did an economic activity last week at least 1 h (YES)	100	73.0
Before been treated, did you work before (YES)	102	74.5
Did you leave the work because of the disease (YES)	85	62.0
Did you experience a reduction on your income because of the disease	97	70.8
During the past 12 months, was there ever a time when your household went without food due to lack of money or other resources? (YES)	86	62.8

#### 3.1.2 Clinical characteristics

Predominantly, the participants were newly admitted patients (*n* = 112, 81.8%), followed by those recovered after dropout (*n* = 10, 7.3%), relapse (*n* = 10, 7.3%), and previously treated patients (*n* = 5, 3.6%). The mean treatment duration was 4.07 months (SD: 3.27, mi*n* = 1, max = 24). A small percentage were healthcare workers (*n* = 6, 4.3%). Over 90% attended the health center for medication, and 95% had not completed treatment in the past 3 months. Patients who could not follow up represent 4.4% (*n* = 6) and 12% (*n* = 17) recovered. Approximately 63% (*n* = 86.3) were self-administering the medication and supervised by video call, 27% (*n* = 37) were self-administering the medication without supervision, 8% (*n* = 10) reported recording a video, and 2.2% (*n* = 3) attended the health center in person (Table 1).

#### 3.1.3 The integrated behavioral model (IBM) categories

We summarized the results in this section. In Table 2 we integrate qualitative and quantitative data.

**Table 2:** Knowledge, attitudes, practices of individuals with TB related to the use of digital technologies for prevention and control, Santo Domingo, 2019 (*n* = 137).

Variables	*n* = 137	%
Know disease symptoms		
Cough	100	73.0
Cold	64	46.7
Weight loss	35	25.5
General malaise	30	21.9
Chest pain	15	10.9
lack of appetite	25	18.2
Night sweats	19	13.9
Weakness or fatigue	18	13.1
Shivers	18	13.1
Phlegm	17	12.4
Know if the disease can be cured	133	97.1
Knows how to cure		
With pills delivered to the health center (self-administered)	111	81.0
With pills that are taken under the supervision of health personnel	23	16.8
Resting at home	12	8.8
Taking natural or home remedies	9	6.6
What makes a person not healed?		
When you do not take the medicine daily	135	98.5
When you do not rest	20	14.6
When you do not pray	3	2.2
When you do not eat well	20	14.6
When you drink alcohol	24	17.5
When you use drugs (juka)	18	13.1
Cellphone can be utilized for healthcare purpose	124	90.5
Know how to use basic features of mobile phones		
Sending and receiving text messages (SMSs)	96	70.1
Sending and receiving voice messages	24	17.5
Making phone calls	19	13.9
Making video calls	17	12.4
Using WhatsApp or other chat	17	12.4
Receiving a reminder of their appointments	9	6.6
As medication monitoring (reminder treatments)	11	8.0
Did you feel well-informed about TB?		
Strongly disagree	44	32.1
Disagree	56	40.9
Neither agree nor disagree	16	11.7
Agree	18	13.1
Strongly agree	3	2.2
Would like to receive health-related text messages over the phone regarding how to keep healthy? (Yes)	115	83.9
Would like to receive reminders over the phone about when to take TB treatment?		
Absolutely appropriate	56	40.9
Appropriate	69	50.4
Neutral	4	2.9
Inappropriate	2	1.5
Absolutely inappropriate	6	4.4
Preference on follow-up by providers for treatment adherence monitoring		
Visiting the TB unit	35	25.5
Supervision at home by the health promotor	48	35.0
Supervision at home by a family member	15	10.9
Sending daily a voice message	14	10.2
Sending daily a text message	4	2.9
Record a video and send it to health personnel	10	7.3
Make a video call with provider	7	5.1
Other	4	2.9
Fear of loss of privacy by utilizing WhatsApp or sending recorded videos to providers		
Very untrue of me	4	2.9
Untrue of me	5	3.6
Neutral	2	1.5
True of me	3	2.2
Very true of me	123	89.8
Preference for using a mobile app (different from WhatsApp) that is more private and confidential for follow-up by providers	74	54
Practices: Reported having a personal cell phone.	121	88.3
Had used their phones		
Several times a day	119	86.9
Used it only once today or in the last 24 h	3	2.2
They do not currently use it.	15	10.9
Purposes of smartphone use		
Chatting (WhatsApp)	68	49.6
Social networks (Facebook, Twitter)	30	21.9
Personal calls	86	62.8
Work calls	14	10.2
Search of products and services	8	5.8
Buy at pharmacies, shopping malls, others	9	6.6
As an alarm clock	7	5.1
Most difficult regarding taking pills (TB treatment)		
Adverse effects	84	61.3
Having something to eat before	36	26.3
Taking it everyday	10	7.3
Comply with an schedule / time	17	12.4
Remind it—always forget	2	1.5
My work	1	0.7

##### 3.1.3.1 Knowledge

Most participants recognized cough (73.0%, *n* = 100) as a disease symptom. Other symptoms were less known such as cold (46.7%, *n* = 64), weight loss (25.5%, *n* = 35), phlegm (12.4%, *n* = 17), shivers (13.1%, *n* = 18), fatigue (13.1%, *n* = 18). Most (97.1%, *n* = 133) know the disease can be cured. Of those, 81.0% (*n* = 111) believe it is cured with pills from the health center (self-administered), 16.8% (*n* = 23) mention that pills should be taken under medical supervision. Some mention resting (8.8%, *n* = 12) and natural or home remedies (6.6%, *n* = 9). Concerning factors preventing recovery, 98.5% (*n* = 135) believe that not taking medicine daily is the main reason. Other factors mentioned include alcohol consumption (17.5%, *n* = 24), poor nutrition (14.6%, *n* = 20), lack of rest (14.6%, *n* = 20), and drug use (13.1%, *n* = 18). Only 2.2% (*n* = 3) believe that lack of prayer affects healing. Table 2 shows descriptive data on knowledge questions.

##### 3.1.3.2 Attitudes

The majority, 90.5% (*n* = 124), understood that a cell phone can be utilized for healthcare purposes. Receiving health-related text messages was highly rated as potentially helpful (84%). Most participants found it appropriate (*n* = 69, 50.4%) or very appropriate (*n* = 56, 40.9%) to receive reminders about their TB treatment on their phone. Regarding their level of information about TB, only 32.1% (*n* = 44) felt ‘very informed’, (40.9%, *n* = 56), while others ranged from somewhat informed (*n* = 16, 11.7%), ‘little informed’ (13.1%, *n* = 18), to ‘not at all informed’ (*n* = 3, 2.1%). Nearly all users understand the importance of taking medication daily to cure the disease (Table 3).

**Table 3:** Merge results from quantitative and qualitative analysis of IBM variables measured in individuals with TB, Santo Domingo, 2019.

Qualitative results	Quantitative results	Merge/integrating results
Knowledge about digital health Knowledge about how technology could be useful for health purposes.	Knowledge about mobile health Limited understanding of TB symptoms Only 16% knew that medication intake must be under health personnel supervision. Participants considered resting at home (12.0, 9%) and traditional remedies (9.0, 7%) were healing methods. About 13% ignored treatment duration.	Most patients have basic knowledge of the use of mobile phones. There is a need to close the knowledge gap and misconceptions. Secure sources of information were not mentioned.
*Positive attitudes* toward remote patient monitoring Recognition of the benefits of treatment and digital health (instrumental—cognitive attitude). It is a relief to not have to attend the program in person. Not worried about using mobile phones for treatment monitoring (experiential—affective attitude) Not being scared about the potential risk of harm due to loss of privacy. *Negative attitudes* toward remote monitoring as a daily activity, a patient manifested that he could send one or two videos per week but not daily. Descriptive norm A patient will commit to sending videos if the doctor requests it. *“If they tell me to send a video taking the pill, from now on I will send it” (FGD 2, male, Participant #1).*	Attitudes Only 40% reported feeling well informed about TB. 84% rated it as potentially helpful to receive text messages about how to improve their health. Most participants consider it appropriate to use their mobile phones to receive reminders about medications. 90% indicated they were not concerned about the loss of privacy when using the internet to send recorded videos to health personnel. More than 50% would prefer to use an alternative application to WhatsApp.	Responses in both qualitative and quantitative methods were coherent. Considering they are not well informed regarding the disease Having positive attitudes toward technology because they see the benefits. Not be concerned with loss of privacy Positive about using a mobile App for remote monitoring
Behavioral importance (Theme 7), The phone is a valuable tool for communication. *‘The phone would help me a lot because that way I can communicate with you” (FGD 1, female, Participant #2).*	124 (90.5%) participants understand mobile phones can be used for health care.	Technologies used for health are a valuable tool.
Practices Previous experience with similar technologies. Self-efficacy Participants recorded themselves taking medications and sharing them with doctors. Personal agency -perceived self-control Participants understand they are responsible for their health. *‘I tell him: doctor, I am taking my medication’ (FGD 1, Participant #2)*	Practices 90% reported having a mobile phone for personal use. 70.0% (*n* = 96) use basic features. Taking medications 21.0% (*n* = 29) said that they had stopped taking medication at some point during their treatment. 5.0% (*n* = 7) reported having stopped taking medication for a week or more.	Qualitative and quantitative data confirmed that patients do have access to mobile phones and the internet. reported using basic functions such as calls, video calls, WhatsApp, etc. reported having used a mobile phone for medical purposes.
Perceived facilitators (Theme 7) Mobile phones facilitate healthcare providers and patient communication and monitoring. *“I have it, I use it, mobile phones are very important for everything, in this case … she [the nurse] calls me when I have to come to the unit” (FGD 1 Participant #1).*	Access to the internet in the last 3 months 98% claimed that they accessed the Internet from home.	Internet access could improve communication between health personnel and patients.
Perceived barriers (Theme 8) Lack of cell phone or mobile device. Limited internet access due to cost. *“I do not have one [a mobile phone]” (FGD 2 Participant #2). ‘Now I have the chip cut off because I have not paid it, (FGD 1 Participant #4).* Lack of health insurance and social assistance programs.	Perceived barriers The most challenging aspect of maintaining adherence was the medication’s adverse effects.	Lack of a digital device, limited access to the Internet, or limited experience using cell phone functionalities and the adverse effects were barriers to patient adherence.
Patient preferences Patients might have a preferred treatment schedule or communication way. ‘I do not like to take the pill in the morning because it is heavy. I believe that the important thing is to take it’ (FGD 1 Participant #2).	Patient preferences 35.0% (*n* = 48) reported preferring health promoters supervise treatment compliance, (7.3%, *n* = 10) send a voice message daily or record a video and send it to the health personnel, (5.1%, *n* = 7) make a daily video call.	Patients have preferences regarding how, where, and when to take the medication and be monitored.

##### 3.1.3.3 Practices

A significant majority, 89.8% (*n* = 123) reported having a personal cell phone. Most participants, 86.9% (*n* = 119), used their phones several times a day, 2.2% (*n* = 3) said they used it only once today or in the last 24 h, while 10.2% (*n* = 14) responded that they do not currently use it. Basic features such as sending and receiving text messages were commonly used (*n* = 96, 70%). About 63% (*n* = 86) used their phones for personal calls, and half of them used it for chatting (WhatsApp), 23% (*n* = 32) for social networks (Facebook, Twitter), 10% (*n* = 14) for work calls, 6.6% (*n* = 9) for shopping, and 5% (*n* = 7) as an alarm clock. Having internet access from home in the last 3 months was reported by 98% (*n* = 123). Few reported using mobile phones as a reminder of their appointments or medication monitoring (*n* = 11, 8%).

#### 3.1.4 Clinical and adherence characteristics

According to medical records, 16.1% (*n* = 22) of participants were irregular or nonadherent to treatment. 35% of participants had at least one comorbidity, with diabetes (11.6%, *n* = 16) and HIV (10.9%, *n* = 15) being the most frequent. Participants reported substance use: alcohol use (8.8%, *n* = 12), tobacco use (5.8%, *n* = 8), and drugs (1.5%, *n* = 2).

### 3.2 Qualitative findings

Nine patients participated in the FGDs: the first group comprised 6 users (4 men, 2 women), with ages ranging from 19 to 56 (mean 35.2), and the second group included 3 men, with ages ranging from 23 to 60 (mean 40.6). Participants had a low socioeconomic status, and the level of education was primary. Every week or every 2 weeks, they pick up the medications as scheduled by the team. The qualitative findings centered on eight core themes from IBM. Exemplars for each theme are presented in Table 2.

#### 3.2.1 Theme 1—knowledge

This theme encompassed two categories: *knowledge about digital tools and knowledge about TB as a disease.* Participants described a lack of information about TB and basic knowledge about digital tools. *“For me, it is important [access to technology] because at the beginning of the disease [TB] one does not know anything” (FGD 1 Participant #1).*

#### 3.2.2 Theme 2—experiential and instrumental attitudes

Participants reported not being concerned about using their cell phones for communication and treatment monitoring (experiential-affective attitude). They recognized the benefits of treatment and using digital health tools (instrumental-cognitive attitude). *‘The doctor told me like [sic] I was not taking the pill, but I take it during the week, and I said: “no, but I’ll make a video for you so you can see me taking it”’ (FGD 1 Participant #4).*

#### 3.2.3 Theme 3—Perceived norm

Participants expressed the need to demonstrate their treatment compliance by sending videos or calling health personnel. Some participants noted they would only send a video if requested explicitly by the doctor (injunctive norm—what others expect them to do -). *“If they tell me to send a video taking the pill, from now on I will send it” (FGD 2, male, Participant #1).*

#### 3.2.4 Theme 4—personal agency

Comments reflected perceived self-control and self-efficacy. *‘I tell him: doctor, I am taking my medication’ (FGD 1, Participant #2). ‘I was at a party, yes, but now I am ready for my thing again [to re-start medication]’ (FGD 2 Participant #1).*

#### 3.2.5 Theme 5—practices

This theme included participants’ familiarity and proficiency in *using technology* and *taking medication*. Participants responded as if it were obvious that they knew how to use “technology” (cell phones), popular apps, and social media. *‘I use WhatsApp, Facebook, and Instagram.’ (FGD 1 Participant #4).*

#### 3.2.6 Theme 6—facilitators

Participants generally perceived mobile devices as facilitators that allow interaction with health personnel. *“I have it, I use it, mobile phones are very important for everything, in this case … she [the nurse] calls me when I have to come to the unit” (FGD 1 Participant #1).*

#### 3.2.7 Theme 7—importance of the behavior

Mobile devices are considered necessary, particularly as reminders and communication channels. *‘The phone would help me a lot because that way I can communicate with you” (FGD 1, female, Participant #2).*

#### 3.2.8 Theme 8—perceived barriers

Perceived barriers were associated with the lack of a digital device, limited internet access, or limited experience using cell phone functionalities (e.g., social networks and video calls). Barriers to treatment adherence also included patient preferences regarding the time to take the medication, lack of health insurance, and participation in government assistance programs. *“I do not have one [a mobile phone]” (FGD 2 Participant #2). ‘Now I have the chip cut off because I have not paid it, (FGD 1 Participant #4)*.

### 3.3 Integrating phase

Quantitative data showed a limited understanding of basic aspects of the disease, and qualitative data confirmed the importance that participants place on digital health. Positive attitudes were identified toward being remotely monitored, receiving text messages, and using their phones for health purposes. Also, they stated that they do not have privacy concerns or are scared about the potential risks of losing privacy while using digital technologies. Key findings for the IBM variables are presented in Table 3.

## 4 Discussion

This study aimed to describe patients’ attitudes and behaviors digital technologies for TB control and prevention. According to medical records and patient reports in the study, 16.1% of the patients showed irregular medication taking. This aligns with the National TB Program’s Annual Report 2020 findings, which reported that 11 to 25% of patients were lost to follow-up treatment, depending on their clinical and sociodemographic characteristics (23). However, the percentage of non-adherence to treatment may be even higher in this population. Unfortunately, the report did not provide a specific indicator for treatment adherence. Participants in the study showed a knowledge gap (e.g., only 16% knew the importance of DOT) and expressed a desire for more information about TB and its treatment. This situation partially explains the high rate of lost follow-up and irregular medication taking. A qualitative study of KAP towards TB in Georgia revealed good knowledge among current and former TB patients (32). Consistent with other studies, we found that patients can learn about their condition (33).

Participants expressed a positive attitude towards the benefits of using digital technology for communication with health personnel to support taking treatment and remaining adherent, dispelling fears of privacy loss. Most participants (*n* = 69, 50.4%) wanted to receive text messages providing health tips and reminders. Several other studies have attempted to evaluate evidence for the efficacy of various DAT interventions such as texting, Short Message Service (SMS) reminders, and mobile phone Apps, among other resources, at improving treatment adherence. Different studies have identified modest or no efficacy of SMS in improving treatment adherence (34–36), while other programs using interactive SMS (14, 15) have proven effective in enhancing medication adherence (16).

Iribarren et al. used the Information-Motivation-Behavioral Skills model to design and document the development of the intervention with a multidisciplinary team of researchers, clinicians, administrators, and patients in active TB treatment (37). In the DR, we used an Integrative Behavioral Model to develop a DAT that is informed by a health behavior model that has been found successful at improving health-related behaviors (28).

Participants reported using avoidance strategies, eluding thinking ‘negative things’ about the disease and focusing on positive aspects of treatment (such as taking medication to get well-perceived self-management). Patients’ may have an interest to demonstrate compliance with treatment as perceived norm on how they should behave as users of the healthcare services. These aspects emphasize the need for tailored text messages supporting self-regulation (16) helping them on how to avoid negative thoughts as the integrative model of behavioral prediction can be used for this purpose (38).

Environmental barriers, such as limited access to digital devices and the internet, were identified in low- and middle-income countries (17–19). Cost considerations, particularly the affordability of cell phone services, pose challenges. The average monthly mobile phone plan exceeds the purchasing power of low-income families, as most often people with TB fall into this category. When writing this report, the average low-income family makes less than $675 per month, whereas the average monthly mobile phone plan is $55.00 (39). Mobile phones are needed in order to use health apps that have been proved to be effective for scheduled follow-up visits and improves the probability of follow-up, reducing missing appointments and optimizing patient outcomes (40). So, improving access to technology to patients can be recommended for low-income TB patients in the DR.

Behavioral models have been effectively applied in TB treatment adherence studies using mobile applications (37, 41–43), and similar models have demonstrated success in understanding TB prevention and treatment (15). In a literature review of articles published between 1995 and 2015 on text messaging and mobile phone apps with adherence or compliance and youth/adolescents, only 32% of the identified studies incorporated a theoretical framework in their design (36). Our study was driven by a theoretical model that can be further reviewed, analyzed, or improved for advancing health behavior science.

### 4.1 Limitations

Data collection occurred during the COVID-19 pandemic in December 2020, potentially influencing participant perspectives. The low number of participants in the FGDs and the need to scrutinize psychometric properties within the IBM constructs should be considered.

The COVID-19 pandemic restrictions impacted the number of patients visiting healthcare services and the number of patients who could be invited to the clinics. Therefore, small numbers were invited to the FGD sessions for safety measures and to comply with guidelines.

### 4.2 Conclusions and recommendations

Findings highlight a digital divide, with some users preferring traditional healthcare visits. Interventions should consider patients’ preferences rather than impose new modalities. As most participants already use mobile phones to communicate with healthcare personnel, a VOT intervention is feasible.

## Data Availability

The raw data supporting the conclusions of this article will be made available by the authors, without undue reservation.
